# Priority of Early Colonizers but No Effect on Cohabitants in a Synergistic Biofilm Community

**DOI:** 10.3389/fmicb.2019.01949

**Published:** 2019-08-23

**Authors:** Nanna Mee Coops Olsen, Henriette Lyng Røder, Jakob Russel, Jonas Stenløkke Madsen, Søren Johannes Sørensen, Mette Burmølle

**Affiliations:** Section of Microbiology, Department of Biology, University of Copenhagen, Copenhagen, Denmark

**Keywords:** multispecies biofilms, interspecies interactions, community assembly, priority effects, synergy

## Abstract

The arrival order of different species to a habitat can strongly impact community assembly and succession dynamics, thus influencing functionality. In this study, we asked how prior colonization of one community member would influence the assembly of a synergistic multispecies biofilm community grown *in vitro*. We expected that the prior arrival would confer an advantage, in particular for good biofilm formers. Yet, we did not know if the cohabitants would be impaired or benefit from the pre-colonization of one member, depending on its ability to form biofilm. We used a consortium consisting of four soil bacteria; *Stenotrophomonas rhizophila, Xanthomonas retroflexus, Microbacterium oxydans* and *Paenibacillus amylolyticus*. This consortium has been shown to act synergistically when grown together, thus increasing biofilm production. The results showed that the two good biofilm formers gained a fitness advantage (increase in abundance) when allowed prior colonization on an abiotic surface before the arrival of their cohabitants. Interestingly, the significantly higher number of the pre-colonized biofilm formers did not affect the resulting composition in the subsequent biofilm after 24 h.

## Introduction

Microbial biofilms in nature are often composed of numerous species that directly or indirectly interact with each other. Interspecies interactions have been shown to be particularly pronounced in mixed-species biofilms, where the spatial structure promotes both competitive and cooperative interactions that play a central role in shaping a biofilm community (Hall-Stoodley et al., 2004; Burmølle et al., 2014; Nadell et al., 2016). An important aspect during biofilm assembly, compared to planktonic communities, is that potential cohabitants physically attach and co-aggregate. Interactions between different species have been reported to both facilitate and impede other species from establishing, during biofilm assembly (Christensen et al., 2002; Trautner et al., 2002; Lin et al., 2018). Thus, the order and timing of species arrival to a habitat can strongly impact the way species interact in a biofilm community by altering the success of establishment of late colonizers (Klayman et al., 2009; Fukami, 2015). In this work, our aim was to investigate how prior colonization of a single member would affect assembly of a multispecies biofilm. We used a consortium consisting of four bacteria; *Stenotrophomonas rhizophila, Xanthomonas retroflexus, Microbacterium oxydans* and *Paenibacillus amylolyticus* that were isolated from the surface of a degrading maize leaf (de la Cruz-Perera et al., 2013). This consortium has been shown to act synergistically and increase biofilm formation when grown together in the Calgary device (Ren et al., 2015). We expected that the prior arrival would confer an advantage, in particular to the good biofilm formers of our consortium. Yet, we did not know if the cohabitants would be impaired or benefit from the pre-colonization of one member, depending on biofilm formation abilities. Many other factors can have a significant impact on community assembly, including the environment, dispersal events, and stochastic processes. Here we assessed changes in biofilm assembly caused by species interactions by keeping the environment as stable as possible. All experiments were conducted at ample amounts of nutrients and run for a shorter period of time or under a continuous flow of nutrients and always at a constant temperature.

## Results

**FIGURE 1:**
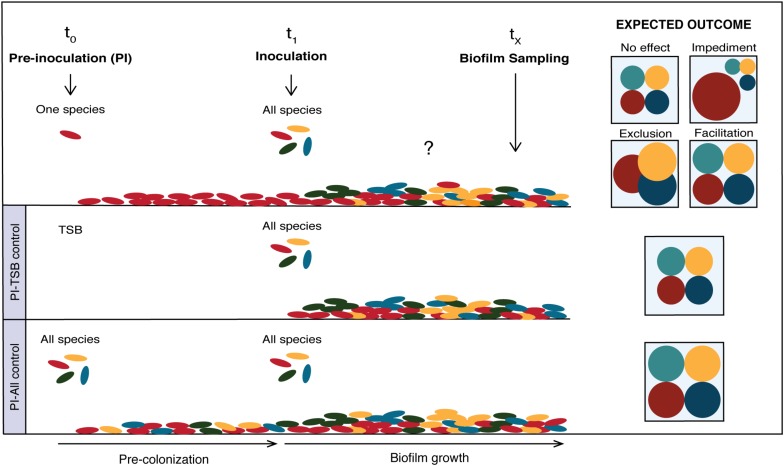
Effect of single-species pre-colonization on subsequent biofilm community composition. Conceptual figure showing hypothetical outcomes of pre-colonization on species compositions. Pre-inoculation with the individual species (top), pre-inoculation PBS control (middle) and pre-inoculation control with all species (bottom). The hypothetical outcomes are depicted on the right in the figure. The effects of pre-inoculation with an individual species could lead to an unchanged species composition (“No effect”) compared to the PBS control, or if the first colonizer has a growth advantage, it could result in “Impediment” or “Exclusion” of cohabitants. The pre-inoculated species could also “Facilitate” the establishment of later colonizers, which would result in increased numbers of the affected species.

The experimental design and hypothetical species compositions of biofilms when pre-colonized with a single species is outlined in Figure 1. First, each of the four species was separately pre-inoculated in the system (t_0_) where it had the opportunity to grow and colonize the glass surface for a certain amount of time. Subsequently, the whole consortium was co-inoculated in the system (t_1_) and let to form a biofilm. The biofilms were sampled and evaluated at different time points (t_X_). Two controls were included for every experiment, “PI-TSB” and “PI-All”, where sterile TSB and inoculums containing all strains were pre-inoculated (PI) in the systems, respectively. Hypothetical outcomes of the effect of pre-inoculation with the individual species, on species composition, are shown in Figure 1. The effects of pre-inoculation could lead to unchanged species composition (Figure 1; no effect), or if the first colonizer has a growth advantage, this could have a negative effect on some of the other species, due to niche preemption (Figure 1; impediment or exclusion). The pre-inoculated species could also facilitate the establishment of later colonizers, which would be seen as an increase in numbers of the affected species (Figure 1; facilitation). As we mentioned, it could be expected that the prior arrival would confer an advantage, in particular to the good biofilm formers of our consortium. We define good biofilm formers as the isolates that can form an adequate biofilm structure on their own. We have added confocal images of the individual species grown alone in Supplementary Figure 1 and they correlate well with results from previous studies (Ren et al., 2015).

### Screening for Effects of Pre-inoculation of Individual Species on Subsequent Biofilm Formation

**FIGURE 2:**
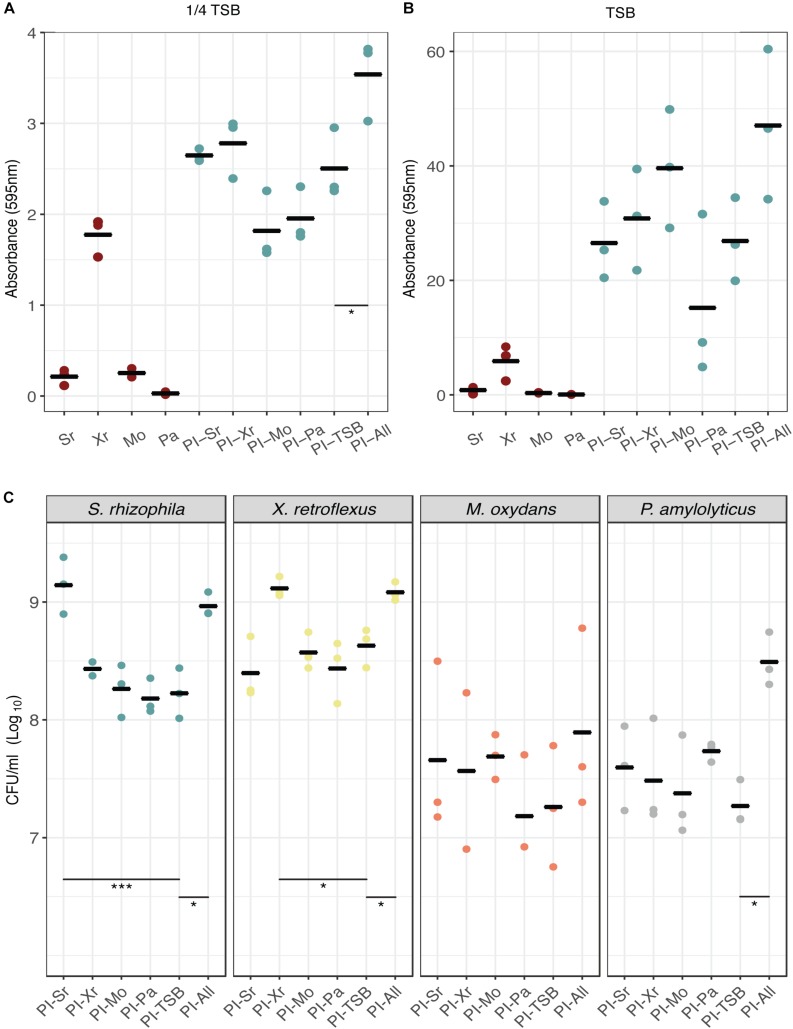
Effect of individual-species pre-colonization on biofilm formation and species composition. Biofilm formation in the Calgary assay was assessed by crystal violet staining after 24 h of growth in 1/4 TSB **(A)** and full strength TSB **(B)**. Mono-species biofilms and biofilms pre-inoculated with TSB or all strains were included as controls. Dunnett’s tests were conducted with PI-TSB as control (single strains were not included), which only showed significant differences between the two controls (*p* < 0.05^∗^) in biofilms grown for 24 h **(A)**. No significant differences were observed in **(B)**. Bars represent means of three biological replicates and dots represent means of four technical replicates (*n* = 3). **(C)** Species enumeration for each pre-inoculation experiment from the DFR. All CFU counts were grouped according to species. Dunnett’s tests were performed within each group with PI-TSB as control. Significant differences between the PI-TSB control and the PI experiments are shown. Bars represent means of three biological replicates and dots represent means of two technical replicates (*n* = 3) (*p* < 0.05^∗^, *p* < 0.001^∗∗∗^).

To initially examine whether pre-inoculation with any of our four species would lead to a change in biofilm assembly, reflected in biofilm production, we used the Calgary assay for pre-inoculation experiments coupled with biofilm quantification by crystal violet staining. We performed the experiments in full strength TSB and 1/4 TSB to test if different nutrient concentrations would affect the outcome (1/4 TSB is still a relatively rich medium and did not impair the growth of the strains). When growing the consortium in 1/4 TSB (Figure 2A) it was found that the synergistic effect was considerably lower compared to using TSB (Figure 2B; Ren et al., 2015). Pre-inoculation with *S. rhizophila* and *X. retroflexus* increased biofilm formation compared to the PI-TSB control (pre-inoculation with 1/4 TSB) and reversely, *M. oxydans* and *P. amylolyticus* pre-inoculation appeared to decrease biofilm formation (Figure 2A). When growing the strains in TSB (Figure 2B), the biofilm production was considerably higher compared to growth in 1/4 TSB. Pre-inoculation with *M. oxydans* seemed to increase biofilm production and pre-inoculation with *P. amylolyticus* led to a decrease in biofilm (Figure 2B). The only significant difference observed (Dunnett’s test) was between the two controls in 1/4 TSB (Figure 2A, PI-TSB vs. PI-All); none of the other tendencies described were statistically supported.

### Early Arrival Conferred a Growth Advantage for Good Biofilm Formers but Did Not Affect the Growth of Their Cohabitants in Biofilms Grown for 24 h in the Drip-Flow Reactor

The initial screen using the Calgary assay suggested that pre-colonization of a single species did not affect subsequent biofilm formation. To further confirm these observations, we used a Drip-flow reactor to estimate species composition in the biofilms. The DFR allows for a continuous supply of nutrients and growth of biofilm on a glass surface under controlled shear forces. The effect of pre-inoculation on community assembly was evaluated by enumerating the individual species in the biofilms (Figure 2C). In the DFR, a significant increase in CFU counts of around 12-fold (*p* < 0.001, Dunnett’s test) for *S. rhizophila* was observed, when it had been pre-inoculated, compared to the PI-TSB control. A similar 10-fold increase (*p* < 0.05, Dunnett’s test) was observed for *X. retroflexus* when this species had been pre-inoculated. The abundances of *S. rhizophila* and *X. retroflexus*, reached the same levels as observed in the PI-All controls, respectively. This growth advantage could be contributed to the fact that they are able to form biofilm alone under the given conditions (Liu et al., 2017, 2018). Surprisingly, this significant increase in *S. rhizophila* and *X. retroflexus*, respectively, did not lead to a change in the abundances of the other members. When *M. oxydans* or *P. amylolyticus* had been pre-inoculated, abundances of these two species appeared to increase compared to the PI-TSB control. The abundance of *M. oxydans* almost reached the PI-All abundance when it had been pre-inoculated, while that of *P. amylolyticus*, as the only species out of the four, did not (Figure 2C). However, these tendencies were not significant (Dunnett’s test), which could also be due to a slightly higher variation in the CFU counts of *P. amylolyticus* and *M. oxydans*. This suggests that good biofilm formers gain a fitness advantage (enhanced prevalence in the biofilm), by arriving first. Notably, without having an effect on the later colonization by the other species.

### Similar Growth Pattern and Spatial Organization of *X. retroflexus* and *S. rhizophila* in Mixed Species Biofilms, Regardless of Prior Colonizers, When Grown in the IBIDI Flow System

**FIGURE 3:**
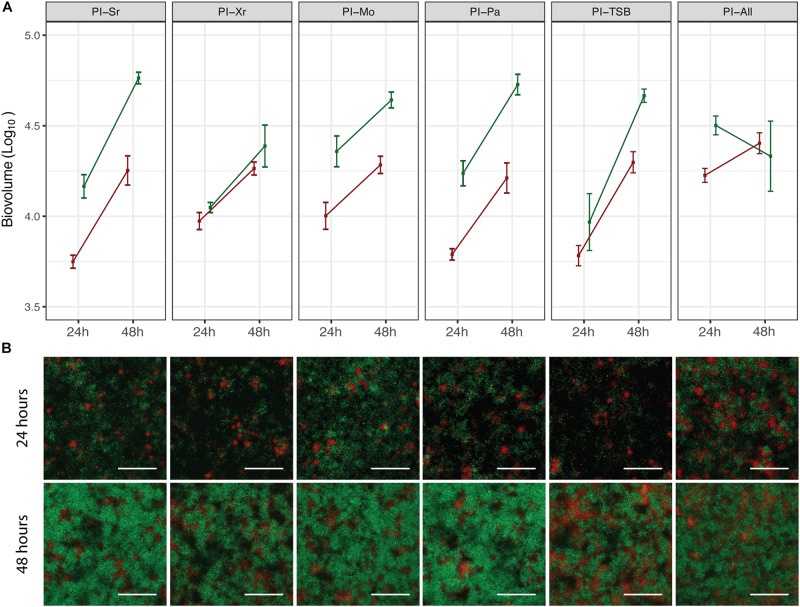
Spatial distribution and quantification of *S. rhizophila* and *X. retroflexus* in mixed species biofilms. **(A)** shows the bio-volume of *S. rhizophila* (green) and *X. retroflexus* (red) at 24 h and 48 h of growth in the respective pre-inoculation experiments, based on pixel quantification of confocal images of the biofilms. Squares and error bars represent means ± SEM. Experiments were performed on three different days and three pictures were acquired per channel (*n* = 3). **(B)** Representative confocal images showing the biofilm spatial organization of *S. rhizophila* (green) and *X. retroflexus* (red) after 24 h (top row) and 48 h (bottom row). From left column to right column: PI-Sr, PI-Xr, PI-Mo, PI-Pa, PI-TSB, PI-All. The depths of the respective biofilms based on the confocal images are (24 h, 48 h): PI-Sr (11 μm, 15 μm), PI-Xr (10 μm, 13 μm), PI-Mo (12 μm, 15 μm), PI-Pa (10 μm, 17 μm), PI-TSB (10 μm, 16 μm), PI-All (12 μm, 18 μm). 40 × magnification. Scale bar indicates 50 μm.

In order to assess if pre-colonization with a specific species had an effect on the spatial distribution of *X. retroflexus* and *S. rhizophila*, pre-inoculation experiments were carried out in the ibidi flow-cell system, which is compatible with confocal microscopy (*M. oxydans* and *P. amylolyticus* could not be stably, fluorescently labeled and were therefore not visually detected). Confocal images acquired from these experiments are presented in Figure 3B. After 24 h, the growth pattern of *X. retroflexus* and *S. rhizophila* appeared similar in all pre-inoculations with *X. retroflexus* (red) located in smaller aggregates spread out on the surface and *S. rhizophila* (green) growing around it in loosely structured clusters (Figure 3B). After 48 h, the cell density of both strains increased, but the growth pattern and spatial localization stayed similar, regardless of the pre-inoculated species (Figure 3B). The bio-volumes of *X. retroflexus* and *S. rhizophila*, respectively, were quantified based on the confocal images (Figure 3A). The quantification supports visual observations from the confocal images (Figure 3B), where both species increase in abundance from 24 h to 48 h. However, when all species had been pre-inoculated (PI-All), *S. rhizophila* biovolume was reduced over time (Figure 3A). As *M. oxydans* and *P. amylolyticus* could not be visually assessed in this setting, we were not able to evaluate their abundances at different pre-inoculation conditions, and it is therefore unknown if results from this ibidi flow-cell system support those of the Drip-flow reactor for these species. These results show that none of the species affected the spatial structure of the subsequent biofilm, when allowed to pre-colonize the surface.

## Discussion

From this study of our synergistic biofilm consortium, we can conclude that mono species pre-colonization did not affect the growth of cohabitants, in the DFR biofilm system. Despite the enhanced growth of early colonizers, no consequential growth reduction was observed for the cohabitants. The significant increase in abundance, observed for the two good biofilm formers, *S. rhizophila* and *X. retroflexus*, and the slightly higher abundances (tendencies) of *P. amylolyticus* and *M. oxydans*, observed when pre-inoculating with those species, indicate that prior arrival to the surface confers a growth advantage that relates to the ability of the pre-inoculated species to attach and form biofilm. It has been observed in other cases that the time of inoculation of different consortium members during biofilm assembly, can greatly impact the success of establishment and thus the species composition. For example, metabolic dependencies and priming of the surface by early colonizers can facilitate other species in biofilm assembly depending on inoculation order (Christensen et al., 2002; Klayman et al., 2009).

These effects caused by early arriving species on later colonizers, also known as priority effects, can be either inhibitory or facilitative (Fukami, 2015), meaning that the history of an assembly can determine the composition, structure, and functionality of a community (Drake, 1991; Chase, 2003). Examples of studies addressing the extent and impact of priority effects include Devervey et al., who reported that strong inhibitory priority effects were observed in mice sequentially infected with pairs of *Borrelia burgdorferi* strains (Devevey et al., 2015). These effects were caused by higher fitness and exclusion by the first administered strain, which resulted in higher transmission of the first strain to ticks. Similarly, Peay et al. found that in a natural community of nectar yeast, negative priority effects were widespread when testing pairwise sequential inoculations of seven flower isolates (Peay et al., 2012). Strong priority effects were correlated with higher phylogenetic relatedness and thus higher metabolic resource overlap. Further, the role of priority effects in the assembly of the gut microbiota in infants was highlighted in a recent review (Sprockett et al., 2018), suggesting that priority effects are important in early life and may strongly influence gut microbiota and infant health.

When growing our consortium in the DFR biofilm system, we observed that an increase in the abundance of a single member in the subsequent biofilm did not affect the abundances of the cohabitants. An explanation to why we do not observe exclusion or impediment by the higher abundance of the pre-inoculated species could be due to their presumed synergistic interactions when grown together. From previous studies, the synergistic effects have been linked to metabolic interactions, including cross-feeding (Hansen et al., 2017; Herschend et al., 2017) and pH stabilization of the local environment by specific members (Herschend et al., 2018). Further, the distinct spatial structure of the different species in the biofilm of this consortium reflected the complex interspecies interactions promoting the synergy observed (Liu et al., 2017, 2018). These synergistic interactions and the fact that we did not see exclusion or a change in the cohabitant composition, when one species increased in abundance, also supports our observations of the spatial organization of *S. rhizophila* and *X. retroflexus* in the mixed species biofilms, that was not affected by prior colonization. However, we cannot exclude the possibility of changes in spatial distribution of *M. oxydans* and *P. amylolyticus* within the biofilm, as these were not visualized.

Overall, we show that in early biofilm assembly of a synergistic consortium, prior arrival to a surface provides a growth advantage to a member, especially if it is a good biofilm former. Also, the increase in abundance of a single member did not affect the cohabitants in the subsequent biofilm. Further, we showed that prior colonization with any individual member, did not impact spatial organization of *X. retroflexus* and *S. rhizophila* within the mixed species biofilms, based on visual evaluation. Our results indicate that priority effects are not prevailing in our synergistic consortium, under the experimental conditions used.

## Materials and Methods

### Bacterial Strains and Growth Conditions

The four bacterial strains *S. rhizophila* (JQ890538), *X. retroflexus* (JQ890537*), M. oxydans* (JQ890539) *and P. amylolyticus* (JQ890540) had previously been isolated from the same soil environment (de la Cruz-Perera et al., 2013; Ren et al., 2014). All strains were kept in glycerol stocks at −80°C and streaked onto Tryptic Soy Broth plates (TSB; 17 g pancreatic digest of casein, 3 g digest of soybean meal, 5 g sodium chloride, 2.5 g dextrose, 2.5 g dibasic potassium phosphate in 1 L distilled water, pH 7.3, Sigma Aldrich, Germany) supplemented with agar (14 g/l, Sigma Aldrich, Germany). Plates were incubated for 2 days at 24°C and grown in TSB overnight, shaking at 250 rpm.

### Construction of Fluorescently Labeled Strains

Constructs of *X. retroflexus* and *S. rhizophila* chromosomally labeled with *mCherry* and *gfp*, respectively, were used in the IBIDI flow-cell setup for confocal imaging. The *X. retroflexus* strain has been described in a previous study (Røder et al., 2018). The construction of *S. rhizophila* was done in a similar way. *GFPmut3* was introduced into the *S. rhizophila* strains using the mini-Tn7 system following the general procedures described by Choi and Schweizer (2006): *S. rhizophila* strains were made electrocompetent by centrifuging (2 min, 10,000 g, 4°C) 1.5 ml overnight culture grown in LB at 30°C while shaking at 250 rpm. Next, the pellet was washed (2 min, 10,000 g, 4°C) three times in 1 ml ice-cold 300 mM sucrose. After a final centrifugation step the pellet was resuspended in 50 μl 300 mM sucrose. The electrocompetent *S. rhizophila* strains were transformed with 25 ng of helper plasmid pTNS2 and 25 ng delivery plasmid pUC18T-miniTn7-P*_*lpp*_GFPmut3*-*Tc*^*R*^. Electroporation was performed using pre-chilled 1 mm gap electroporation cuvettes (BIO-RAD) and a Micropulser electroporation apparatus (BIO-RAD). Rescue and phenotypic expression was performed in pre-heated 30°C SOC broth for 2 h at 250 rpm. Selection was performed on LB agar plates supplemented with 30 μg/ml tetracycline. Insertion of P*_*lpp*_GFPmut3*-*Tc*^*R*^ (via pUC18T-miniTn7-P*_*lpp*_GFPmut3*-*Tc*^*R*^) into the chromosome of the *S. rhizophila* strains was verified by flow cytometry, fluorescence microscopy and the absence of plasmid DNA (Plasmid mini AX kit, A&A Biotechnology).

pUC18T-miniTn7-P*_*lpp*_GFPmut3*-*Tc*^*R*^ was generated in the following way: *GFPmut3 by* was amplified by PCR using primers 5′-GGAGGATTTACATATG CGCAAAGG-3′- (*Nde*I site underlined) and 5′–GTCAAGAAGCTT TGCC TGGCGGCAGTAG-3′- (*Hin*dIII site underlined) with pEntranceposone pEnt-P*_*A*__1__*O*__4__*O*__3_GFPmut3-Km^*R*^* (Klümper et al., 2015) as the template. This PCR product and pUC18T-miniTn7-P*_*lpp*_mCherry*-*Tc*^*R*^ (Røder et al., 2018) were digested with *Nde*I and *Hin*dIII. The two fragments were fused together by T4 ligation, generating pUC18T-miniTn7-P*_*lpp*_GFPmut3*-*Tc*^*R*^, which was transformed into electrocompetent *Escherichia coli* Genehogs (Invitrogen) cells and selected for on LB agar plates supplemented with 15 μg/ml tetracycline.

### Screening for Biofilm Formation Using the Calgary Assay

Biofilm formation was assayed and quantified as previously described by Nunc-TSP lid system and crystal violet (CV) assay based on the Calgary assay (Ceri et al., 1999; Ren et al., 2014). Overnight cultures of the respective strains were re-inoculated in fresh TSB and grown to exponential phase. The cultures were then adjusted to an OD_600__nm_ = 0.1 in 1/4 TSB (diluted in phosphate buffered saline) or full strength TSB and each species was inoculated individually into Nunc-TSP plates (Cat. No. 445497, Thermo Scientific, Hvidovre, Denmark) for 6 h, at 24°C without shaking. The peg lid was moved to fresh media inoculated with all four strains, followed by incubation for an additional 18 h. Controls were included where pre-inoculation for the first 6 h was done with TSB or all strains combined in equal ratios (based on OD). Also, individual species grown for 24 h were included as controls. Biofilm formed on the pegs was washed once in PBS and dried for 5 min, following staining in 1% crystal violet for 20 min. Biofilms were washed 5 times in PBS and de-stained in 96% ethanol. The absorbance was measured at 595 nm using an ELX 808IU Absorbance Microplate Reader (BioTek Instruments, Winooski, VT, United States). The biofilm experiments were repeated three times on three independent days with four replicates each time.

### Biofilm Growth in a Continuous Drip-Flow Reactor System

Biofilms were grown in Drip-flow reactors (DFR) (Goeres et al., 2009; Liu et al., 2017) as described by Liu et al. (2017) (Supplementary Figure 2). Overnight cultures of the four strains, grown in TSB, were diluted to OD_600__nm_ = 0.15 in 50% TSB and 20 ml/channel was used for seeding the bacteria on microscope glass slides (VWR international, China) for 4 h at RT. After seeding the DFR was inclined so that glass slides were slanted in a 10° angle within the reactor to let the medium drip down the entire length of the slide, where biofilm was formed. The flow of media (50% TSB) was initiated at a flow rate of 0.4 ml/min by a peristaltic pump. Each species was allowed to form biofilm for 6 h at 24°C, followed by inoculation of all channels and second seeding with all species for 1 h. The biofilms were then inoculated for an additional 18 h. Biofilms were harvested by placing each slide in a 50 ml falcon tube filled with 20 ml PBS, followed by vigorous vortexing and pipetting to remove all biofilm from the slides. Two controls were included in each experiment; pre-inoculation for the first 6 h with all species and TSB, respectively. Three biological replicates with two technical replicates were conducted.

### Species Quantification

From the biofilm bacterial suspension from the DFR, 10-fold dilution series were prepared in 0.9% NaCl and plated on Congo Red plates (TSA complemented with 40 μg/mL Congo Red and 20 μg/mL Coomassie Brilliant Blue G250) prepared with or without kanamycin (50 μg/ml) in order to select for *X. retroflexus*. *S. rhizophila* and *X. retroflexus* are morphologically similar, however, *X. retroflexus* is resistant to kanamycin, whereas *S. rhizophila* is sensitive. The plates were incubated at 24°C for 2–3 days and stored at 4°C until estimating total CFU counts and species ratios based on colony morphology and kanamycin resistance.

(The statistical analyses were conducted using analysis of variance test followed by Dunnett’s test. *p*-values < 0.05 were regarded as statistically significant.)

### Biofilm Cultivation in ibidi Flow Chambers

In order to evaluate the effect of pre-inoculation in biofilms grown under continuous flow, we used the commercially available ibidi μ-Slide^VI^ ^0.4^ flow chambers (ibidi GmBH, Germany). The ibidi flow-cells have a similar setup as the DFR, however, the flow chambers are filled with media, to keep a continuous flow that allows for a stable nutrient level and shear stress within the chambers. for A 5 l media bottle, inverted to avoid bubbles in the system, was connected to the inlets of the flow chamber via silicone tubes and the outlet tubes were attached to waste bottles via a peristaltic pump (Watson-Marlow, Falmouth, United Kingdom). The experimental procedure was similar to when screening for biofilm formation in the Calgary device. Briefly, cultures in exponential phase were diluted to an OD_600__nm_ of 0.15 in 25% TSB. 500 μl/channel was used for inoculation of the flow chambers, through injection ports (ibidi GmBH, Germany). Each species was seeded for 1 h at RT, in separate channels with arrested flow, after which, the flow was initiated at 1 rpm which corresponds to a rate of 42 ul/(min × channel), for 6 h at RT. The flow was paused again and fresh cultures of all four species were co-inoculated for 1 h in all channels. The biofilms were then grown for a total of 24 h or 48 h before evaluation. Pre-inoculation with TSB or co-culture for the first 6 h were included as controls for each experiment.

### Confocal Image Processing and Analysis

Image acquisition was performed with a CLSM (LSM800, Zeiss, Germany) equipped with a 40 × objective. Images were acquired from the part of the biofilm closest to the inlet (Supplementary Figure 3) with excitation wavelengths at 561 and 488 nm applied for mCherry and GFP, respectively, while maximum emission wavelengths for mCherry and GFP were 610 and 509 nm, respectively. Based on our own observations and previous studies (Lee et al., 2014), biofilms grown in flow-cells display a high degree of spatial heterogeneity. The channels of the CLSM images were separated and the red and the green channel were converted to 8-bit in Fiji (Schindelin et al., 2012). The images were converted to.tiff files and were further processed in the R statistical language (R Core Team, 2017) using in-house R scripts for image preparation and biomass quantification as previously described (Liu et al., 2017). Scripts are available as an R package on GitHub^1^. Otsu’s method was used for thresholding. All images were smoothed before quantifying biomass. The confocal images used for visual evaluation were converted to.tiff files and further processed (denoise and median) using the software Zen lite (Zeiss, Germany).

## Author Contributions

NO and HR performed the experiments. NO wrote the draft manuscript. All authors discussed the results and commented on the manuscript.

## Conflict of Interest Statement

The authors declare that the research was conducted in the absence of any commercial or financial relationships that could be construed as a potential conflict of interest.

## Footnotes

1 www.github.com/Russel88/RCon3D, v1.2.2.
