# Supplementary material for: Priority of Early Colonizers but No Effect on Cohabitants in a Synergistic Biofilm Community
Source: Front Microbiol. 2019 Aug 23;10:1949. doi: 10.3389/fmicb.2019.01949 (PMC6716445; doi:10.3389/fmicb.2019.01949)
Supplement: Supplementary file 1 [file Table_1.docx]

**Supplementary Material**

**Supplementary Figures**

**Supplementary Figure 1:** **Individual species biofilm formation in the ibidi flow-cell system.** Confocal microscopy images of single species biofilms after 24 h, 48 h and 96 h of growth under continuous flow. *S. rhizophila* (GFP tagged) and *X. retroflexus* (mCherry tagged) show biofilm formation after 48 h (images of *S. rhizophila* after 24 h was unfortunately not obtained) and 24 h of growth, respectively. *M. oxydans* and *P. amylolyticus* only attach to the surface and form very small aggregates at the side of the flow chamber, respectively. 40x magnification. Scale bars represent 20 μm.


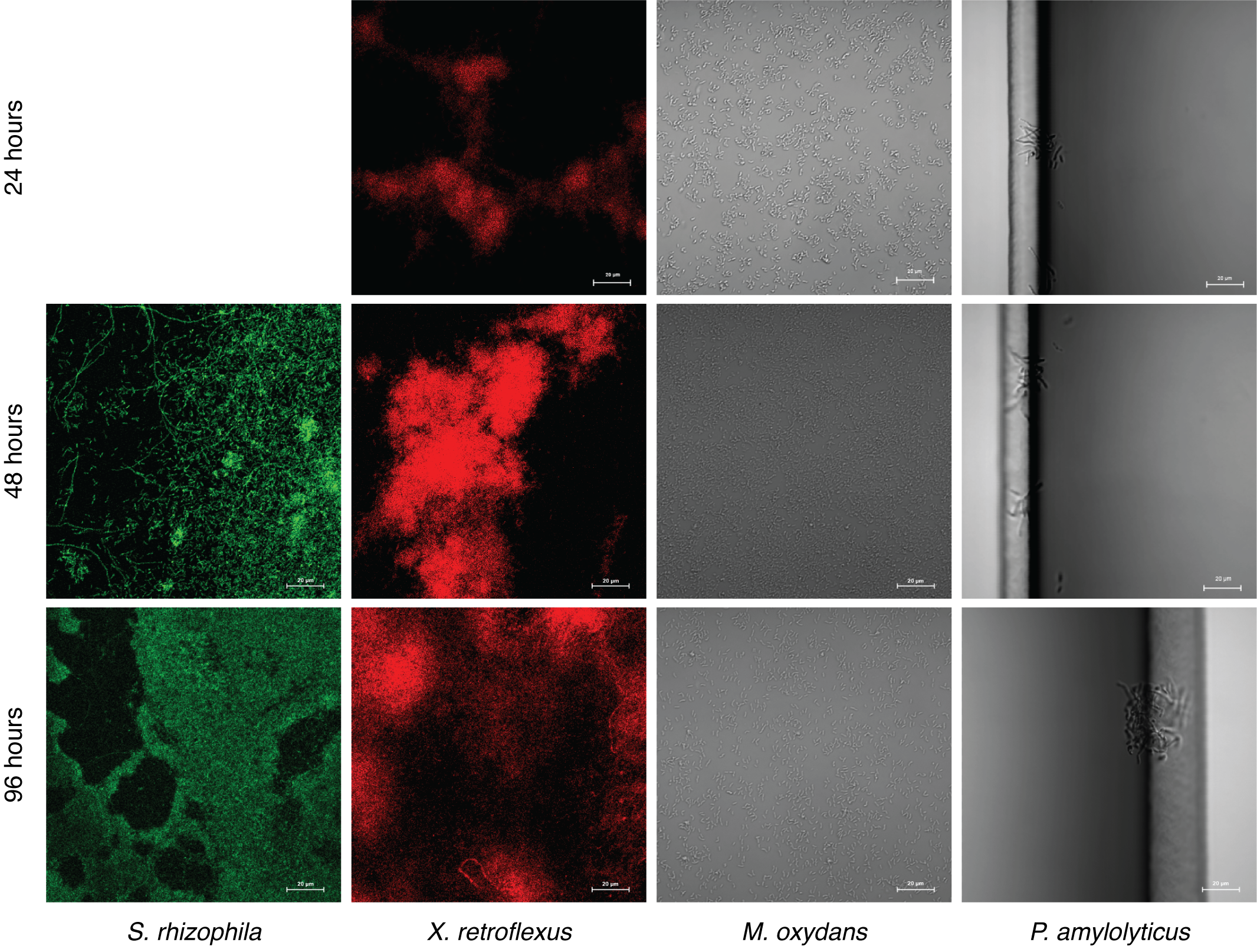


**Supplementary Figure 2: Drip-Flow Reactor setup.** (**A**) Shows the entire setup with media bottles, peristaltic pump, flow chambers and waste bottles, all connected by silicone tubing. (**B)** and (**C**) are close-up of the flow chambers and in image (**C**) it can be noticed how the chambers are slanted in order for the media to drip from the inlets (right) and flow across the glass slide inside the sterile chamber, where the effluent is removed to waste bottles via silicone tubing (left). (D) Shows a graphical representation of the flow chambers.


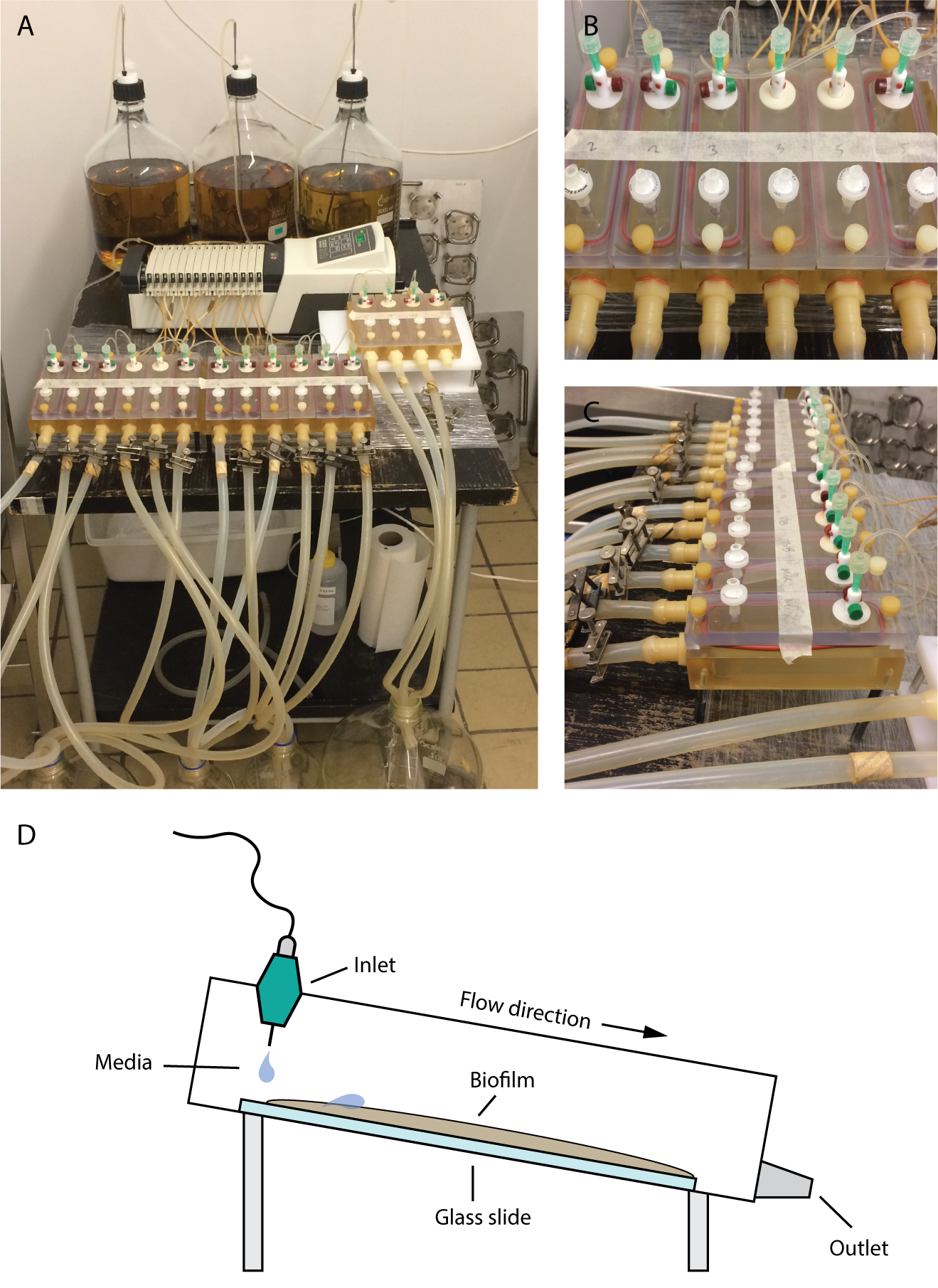


**Supplementary Figure 3: Graphical overview of ibidi flow-cell channel and sampling area for confocal image analysis.**
